# A rare HCN4 variant combined with sick sinus syndrome, left ventricular noncompaction, and complex congenital heart disease

**DOI:** 10.1080/19336950.2025.2517851

**Published:** 2025-07-04

**Authors:** Fengxiao Zhang, Ning Zhao, Lin Wang, Hua Peng, Ying Jiang, Min Cheng, Feng Zhu

**Affiliations:** a Department of Cardiology, Union Hospital, Tongji Medical College, Huazhong University of Science and Technology, Wuhan, China; b Clinic Center of Human Gene Research, Union Hospital, Tongji Medical College, Huazhong University of Science and Technology, Wuhan, China; c Pediatric Department, Union Hospital, Tongji Medical College, Huazhong University of Science and Technology, Wuhan, China

**Keywords:** Sick sinus syndrome, left ventricular noncompaction, HCN4

## Abstract

The hyperpolarization-activated cyclic nucleotide-gated potassium channel 4 (HCN4) gene has been reported to regulate the spontaneous depolarization of sinoatrial node cells. A novel HCN4 mutation (c.2036 G>A) may lead to sick sinus syndrome. The green fluorescent protein (GFP) and either the wild-type (WT) or C679Y mutant (mut) were co-transfected into HEK293 cells to investigate the impact of the mutation on HCN4 channel function. The whole-cell patch-clamp approach was utilized to record HCN4 currents. According to electrophysiological recording, the current amplitude and density generated by mut-C679Y HCN4 channels were much lower than those generated by WT channels. HCN4 channel current activation was not significantly affected by the C679Y mutation. Because of the little current, analyzing the mut channel deactivation kinetic was challenging. Thus, we have identified a novel HCN4 gene mutation that is connected to bradycardia, left ventricular noncompaction, and diverse valve-related heart conditions.

## Introduction

Some cardiomyocytes such as cardiac conduction system, have the property of automatically producing rhythmic excitation in the absence of external stimulation, which is called automaticity. Phase 4 diastolic depolarization, or normal automaticity, is characteristic of the auto depolarization of self-regulatory cells, provides the electrophysiological basis for this self-regulatory action. Funny current (I_f_) channel activation contribute to phase 4 depolarization, and spontaneous activity of pacemaker cells [1].

The ion currents involved in each depolarization of SA node cells include: If, T-type transient calcium (ICa-T), L-type transient calcium (ICa-L), intermediate conductance potassium (IK) current and sodium-calcium exchange current (NCX), etc [2]. However, among many ion currents, the I_f_ current passing through the If channel is the most important, so it is called “pacemaker current” [1]. The I_f_ channels can be activated by electricity or chemicals. It is activated at the hyperpolarization of the cell membrane (−50~-70 mV) and fully activated at −100 mV. The current increases with an increase in hyperpolarization. The I_f_ current is composed of multiple cation flows (anions cannot pass through the If channel), and the mixed permeability of Na^+^ and K^+^ is the characteristic of the If channel, that is, under physiological conditions, the I_f_ current is mediated by Na^+^ and K^+^. When the I_f_ current is open, there will be more Na^+^ inflow and a small amount of K^+^ outflow to form the I_f_ current, and then start the slow automatic depolarization of the autonomic cardiomyocytes in phase 4. The I_f_ current can also be activated by intracellular cyclic adenosine phosphate (cAMP) [3]. The cAMP is an important second messenger in the process of biological signal transduction. It is purported that the effect of cAMP on the I_f_ channel is not related to the process of basic phosphorylation, but directly affects the I_f_ channel. The cAMP regulates I_f_ current, enhances the opening of HCN channels, accelerates its activation, and slows deactivation by shifting the activated voltage dependence to more depolarization potentials [3].

Hyperpolarization-activated cyclic nucleotide-gated (HCN) channels are encoded by the HCN1–4 gene family. The HCN4 gene regulates the spontaneous diastolic membrane depolarization of SA node cells [4,5]. It is composed of 1203 amino acids, including 6 transmembrane segments (S1-S6), a pore region, and a cyclic C-terminal nucleotide-binding domain (CNBD). Mutations in this gene have been linked to atrial arrhythmias, sinus bradycardia, and atrial fibrillation [5,6]. It has also been reported that HCN4 is associated with left ventricular noncompaction cardiomyopathy (LVNC) [6,7].

Herein, we describe a patient with sinus bradycardia and rare cardiac anomalies, such as quadricuspid aortic valves (QAV), multivalued pulmonary valves LVNC, and ventricular septal defect. A novel HCN4 variant was also identified and the voltage clamp was performed to test the function of rare mutation of HCN4.

## Results

### Clinical characteristics

**Figure 1.**
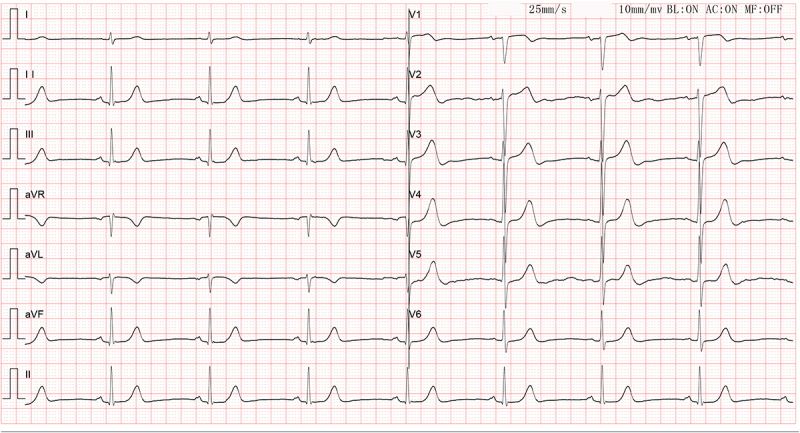
Electrocardiograph of the proband. The ECG showed sinus bradycardia with a heart rate of 47 beats.

The proband, a 15-year-old male, was admitted for chest pain. The electrocardiogram (ECG) showed sinus bradycardia (Figure 1). Echocardiographic investigation and ECG of his parents did not reveal signs of cardiomyopathy and arrhythmia.

**Figure 2.**
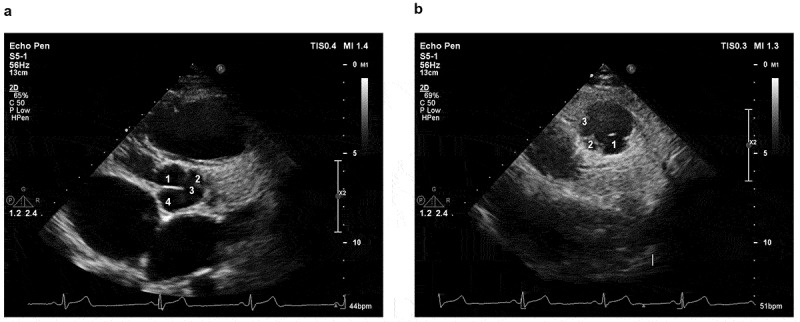
Echocardiographic image of the patient with combined malformation of aortic valve and pulmonary valve. (a). Short-axis transesophageal echocardiogram (TEE) image shows four cusps of the aortic valve. (b). Short-axis TEE image shows more than three cusps of the pulmonary valve.

**Figure 3.**
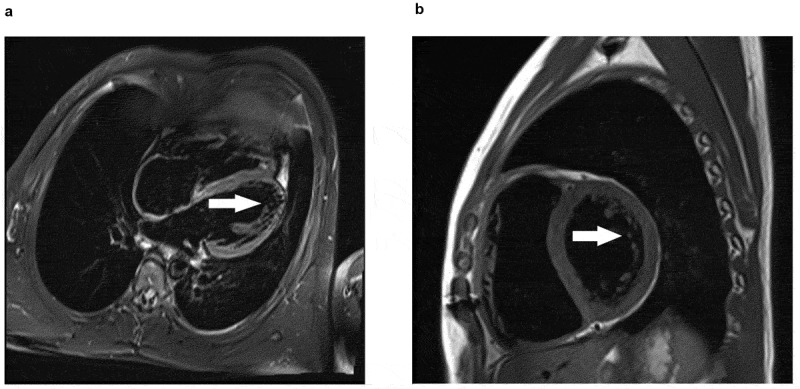
Cardiac magnetic resonance of the patient with LVNC. Fat-suppressed T2-weighted imaging shows a non-compacted myocardium.

The transthoracic ultrasound of the heart showed malformation of both the aortic and quadricuspid aortic valve (QAV) (Supplemental Video 1) and multivalued pulmonary valves. The aortic valve had three nearly equal-sized cusps and one larger cusp, the stenosis. The pulmonary valve also had more than three leaflets, with severe insufficiency and mild stenosis. The right atrium and ventricle were severely enlarged, while the left ventricle and atrium were slightly larger (Figure 2). The cardiac magnetic resonance showed ventricular noncompaction at basal anterolateral, mid-lateral, mid-inferolateral, mid-anterolateral, and apical segments of the left ventricle (Figure 3).

### Identification of candidate variants

**Figure 4.**
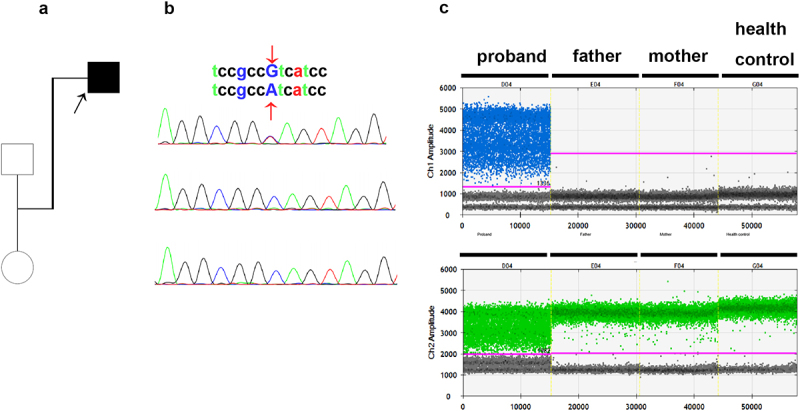
(a). Family tree of the proband. Squares represent male family members and circles represent female family members. The proband is represented by a black square. (b). Sanger sequencing validation of the HCN4 mutation in blood samples. The C679Y mutation is denoted by the red arrowheads. Samples are numbered as follows: 1, proband; 2, father; and 3, mother. (c). Fluorescence intensity of the droplets following amplification of the HCN4 mut region using the ddPCR expert design assays (Bio-Rad laboratories). The four ddPcrs are divided by vertical dotted yellow lines for the proband, mother, father, and health control. The pink line is the threshold, above which are positive droplets (blue and green), and below which are negative droplets (gray) without any target DNA. There is no target DNA for the mutant locus c.2036 G>A in the mother and father.

Genetic analysis was performed on the proband to explore the genetic etiology of the overlapping cardiac disorder in the family. The pedigree tree is shown in Figure 4a. Copy number variation sequencing (CNV-seq) analysis showed that no structural variants (SVs) were related to either congenital heart disease or arrhythmia at the whole-genome level (Supplementary Table S1). Trio-whole exome sequencing (WES) was performed on the family trios to further determine the potential genetic cause of cardiac abnormalities. Using the human reference genome (GRCh37/hg19), 89021 variations were detected through WES, comprising 76,151 SNVs and 12,870 indels. After filtering, 245 variants across 87 genes remained. These genes were prioritized by the Phenolyzer based on input Human Phenotype Ontology (HPO) terms related to heart abnormalities, with HCN4, identified as the top candidate gene. The identified variant in the HCN4 gene was a heterozygous G-to-A transition NM_005477.3:c.2036 G>A, which leads to the substitution of cysteine to tyrosine at codon 679 (NP_005468.1:p.Cys679Tyr) in the cyclic nucleotide-binding domain of the channel protein. This particular variant is not found in commonly used population databases such as the Genome Aggregation Database, 1000 Genomes, and Exome Aggregation Consortium database. It is classified in ClinVar as a variant of uncertain significance linked to Brugada syndrome (Variation ID: 959409). So far, no cases of this variant in individuals with HCN4-related conditions have been documented in the literature. Functional impact assessments on the channel protein suggest that this variant is considered damaging and pathogenic by Sorting Intolerant From Tolerant (SIFT) (score = 0), MutationTaster (score = 1), clinPred (score = 0.998), and Polyphen2 (score = 0.997).

Both WES and Sanger sequencing found that the HCN4 variant c.2036 G>A/p.Cys679Tyr was present in the proband but absent in his unaffected parents, suggesting the *de novo* nature of the variant (Figure 4b). The *de novo* variants may be low-level inherited parental mosaic variants. Subsequently, ddPCR was additionally performed on the blood samples of the family trios to rule out parental somatic mosaicism of the HCN4 variant. Using sensitive ddPCR, no evidence of parental mosaicism was found in parental blood samples. However, 50.5% of the mut-HCN4 allele (c.2036 G>A) was detected in the proband’s blood samples; this allele was not present in the parental blood samples (Figure 4c).

### Electrophysiology of mut-HCN4 channels

Half of the patients with HCN4 variant-causing SSS displayed the cardiomyopathy phenotype of LVNC. However, the mechanism underlying the overlapping disorder is less understood. The C679Y mutation is located in the cytoplasmic CNBD domain of the HCN4 channel. Previous studies found that mutations in the CNBD domain of HCN4 reduced pacemaker current [6,8].

To elucidate the mechanism by which the C679Y mutation impacts the function of the HCN4 channel, HEK293 cells were simultaneously transfected with either the WT or C679Y mut along with GFP. The activity of HCN4 currents was then measured using the whole-cell patch-clamp technique as previous [9].

**Figure 5.**
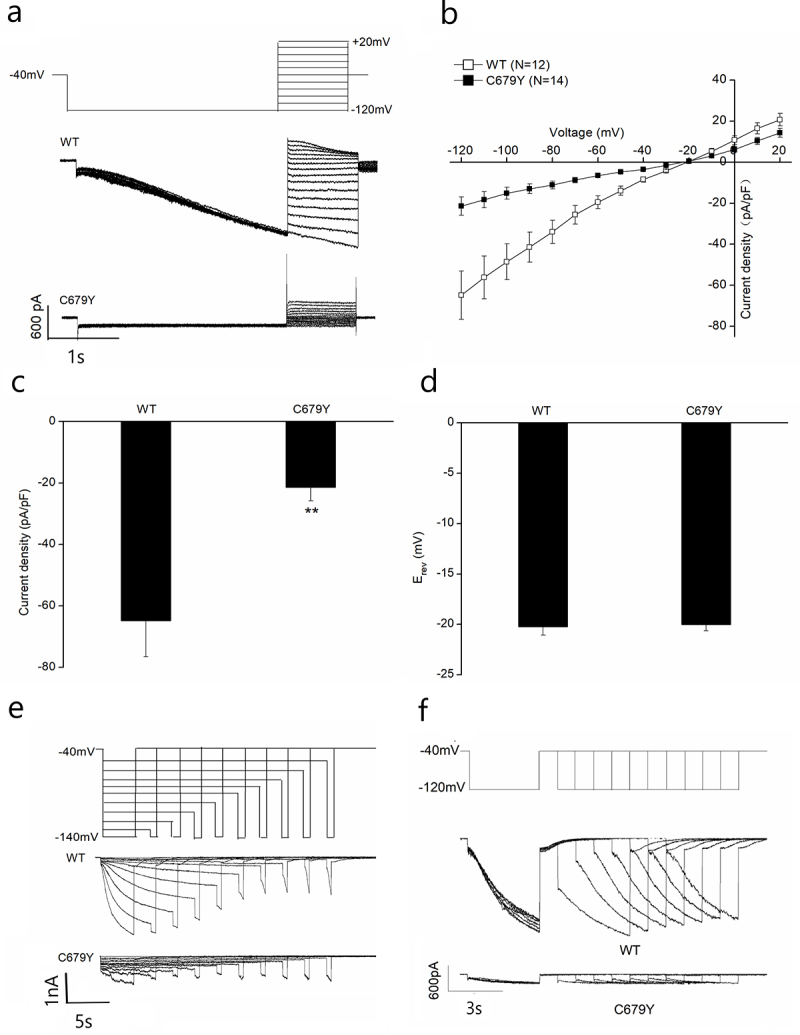
I-V relationship and activation and deactivation properties of C679Y. (a). Voltage protocol and representative current traces were recorded from HEK293 cells expressing wild-type (WT) and C679Y HCN4 channels. (b). Resulting current density amplitude is plotted against voltage. (c). Current densities recorded at −120 mV membrane potential. (d). Reversal potential mean values for WT and C679Y channels. The reversal potential was computed utilizing a third-order polynomial function. (e). Activation pulse protocol (above) of the activation curve and the representative whole-cell current traces of HEK293 cells expressing HCN4. (f). Deactivation pulse protocol (above) and the representative whole-cell current traces of HEK293 cells expressing WT and C679Y HCN4 channels. T two-tailed unpaired Student’s t-test was used for the statistical difference analysis.

Functional effects of the HCN4-C679Y mutation (mut-HCN4) were studied in HEK293 cells using the whole-cell patch clamp technique. For measurement of the I-V relationship, cells were transfected with equal amounts of plasmid DNA encoding for WT- and mut-HCN4 subunits. A first step to −120 mV was made to open the majority of channels, followed by steps to different potentials. The representative current traces of WT- and mut-HCN4 channels are shown in Figure 5a. Current densities of mut-HCN4 were significantly decreased compared to those of WT-HCN4 from −120 mV to −40 mV (Figure 5b). Quantification during single voltage pulses to −120 mV yielded WT-HCN4-mediated current densities of −64.8 ± 11.8 pA/pF (*n* = 12) compared with −21.4 ± 4.4 pA/pF (*n* = 14) for mut-HCN4 (Figure 5c, *p* < 0.01). Reversal potentials (E_rev_) of WT- and mut-HCN4 channels were not significantly different, indicating that they have similar ion selectivity (Figure 5d). The representative current traces of WT and mut-HCN4 channels are shown in Figure 5e. The deactivation pulse protocol and the representative whole-cell current traces of WT and mut-HCN4 channels are shown in Figure 5f. The deactivation properties of mut-HCN4 channels were difficult to analyze due to the very small current.

## Discussion

The present study reports a patient with QAV and multivalued pulmonary valves. The genetic testing revealed a novel *de novo* heterozygous missense HCN4: c.2036 G>A/(p.Cys679Tyr). The whole-cell patch-clamp technique demonstrated that this variant led to the inhibition of HCN4 channels. It was inferred that this novel HCN4 variant is associated with the clinical presentation of bradycardia, LVNC, and multiple valvular heart disease.

QAV is very rare, accounting for < 0.05% of all heart diseases. Most cases of QAV are identified by preoperative two-dimensional echocardiography or arteriography. QAV is caused by valve dysplasia in the embryonic stage. In the early embryonic stage, valve dysplasia is caused by dysplasia of the intimal eminence of the arterial trunk during valve formation. Unlike quadricuspid pulmonary valve malformation(QPV), QAV variant is less likely to cause valve dysfunction. QPV-caused valve dysfunction is more common, and about 44% of patients have valve dysfunction [10]. Except for the HCN4, the present study found no pathogenic mutations of known disease genes that contribute to cardiac dysplasia; numerous teratogenic factors were also excluded, including infectious agents, physicochemical agents, drugs, and certain maternal conditions. It was therefore inferred that the HCN4 variant c.2036 G>A/(p.Cys679Tyr) is associated with the QAV and multivalued pulmonary valves.

**Figure 6.**
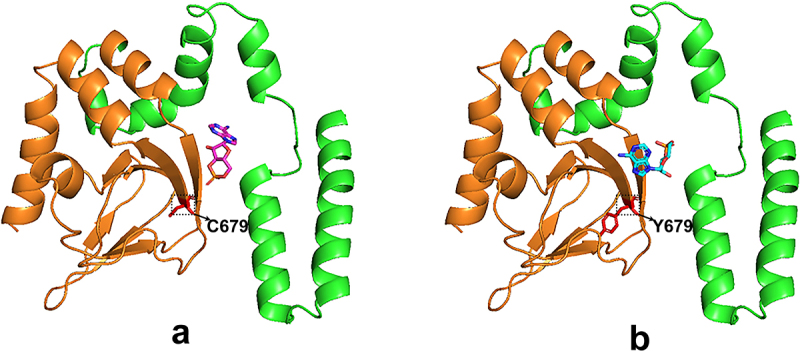
Crystal structure of the WT-HCN4 (a) and mut-HCN4 (b) proteins predicted by software Alphafold 2. The amino acid residues from position 521 to 713 of the HCN4 protein are marked in green. The region (the 602nd to the 720th amino acid residues) encompasses the cyclic nucleotide-binding domain (CNBD), which is marked in brown. The 679th amino acid residue is highlighted in red, which is cysteine in wt-HCN4 (a) and tyrosine in mut-HCN4 (b). The small molecule represents cAMP.

The novel HCN4 mutation was first identified in the patient affected by the combined phenotype. Most arrhythmogenic mutations reported so far in the HCN4 channel are loss-of-function, mostly associated with bradycardia [11]. Previous reports mainly focused on the association between HCN4 gene mutations and sick sinus syndrome (SSS) [2–4]. Some studies suggest that HCN4 May cause sinus bradycardia and left ventricular myocardial compaction [5]; these two clinical abnormalities are caused by the common HCN4 gene mutation. Different from the mutation of the HCN4 protein p.Gly482Arg discovered by Wacker-Gussmann et al [12], this study found that this patient had objective clinical evidence of sinus bradycardia and multivalued pulmonary valves, with a mutation of p. Cys679Tyr carrying the HCN4 protein (Figure 6).

Previous experiments showed that the regulation of cAMP on the HCN channel did not depend on phosphorylation but enhanced diastolic I_f_ inward current through the combination of the cAMP molecule and the CNBD region at the C-terminal of the HCN channel, thus accelerating the rate of phase 4 depolarization and improving self-discipline. In this study, a mutation of p. Cys679Tyr inhibited the regulation effect of cAMP on the HCN4 channel.

Among previously documented arrhythmogenic mutations, loss-of-function HCN4 mutations involving C-terminus residues (such as D553N, K530N, and S672R), involve an alteration in electric charge. Additionally, several studies suggest that the distribution of electric charge in the C-terminus affects channel gating [13]. As previously reported, the C679 residue is located in the CNBD of the HCN4 channel [14]. Our data indicated that the C679Y mutation reduced the channel current but did not change the intrinsic voltage dependency of channel activation in the absence of cAMP. Thus, the current decrease may be because the C679Y mutation affected HCN4 channel potency to cAMP. In addition, when cAMP binds to the channels during the resting state, a neighboring mutation of S672R decreases this binding, dramatically increasing the unbinding rate following channel deactivation [15]. S672R mutation was found in patients with severe sinus bradycardia, which resulted in an average 29% drop in heart rates [16,17]. Contrary to our findings regarding the C679Y mutation, the initial electrophysiological assessments of the hHCN4/S672R mut channel showed a decrease in the voltage required for gating [17]. These studies highlight the important role of the structural element mutation in stabilizing the bound ligand at the binding pocket. However, cAMP was not explored further in the present study, partly because of the small mut current. Hence, future studies should investigate channel HCN4 mutation C679Y potency to cAMP.

Furthermore, our findings demonstrated that the patient had ECG manifestations of bradycardia. Meanwhile, the patient was accompanied by dizziness, and certain physiological and drug effects were ruled out, which was in line with the diagnosis of chronic SSS. Therefore, the HCN4 gene should be listed as a genetic screening for familial SSS.

In addition to the electrophysiological aspects of the heart, the patient also had structural problems with the heart. The echocardiography showed multiple valvular diseases, and magnetic resonance imaging revealed LVNC. LVNC is commonly associated with some complex congenital cardiac anomalies, including the bicuspid aortic valve [18]. We speculate that HCN4 mutations may cause the combined clinical presentation of bradycardia and structural problems of proband.

Previous studies have only suggested that HCN4 is related to arrhythmia or heart failure, and this study is the first to find that HCN4 defects can cause simultaneously the clinical presentation of bradycardia, LVNC, and multiple valvular heart disease. It is suggested that the role of this gene in heart development needs to be further studied and explored.

## Materials and methods

### Clinical evaluations

The study was in accordance with the Declaration of Helsinki and was approved by the Ethical Committee of Tongji Hospital, affiliated with Tongji Medical College at Huazhong University of Science and Technology in Wuhan, China. Prior to entering the study, each subject will be provided with a properly executed written informed consent (Minors shall have their informed consent signed by their guardian). All participants submitted written informed consent to collect and publish their information. All efforts to anonymize individuals were taken.

### Genetic testing, sequencing, and bioinformatic analysis

The peripheral blood was collected from a proband and his parents. Low-coverage (3X) whole-genome sequencing (WGS) was carried out on the proband to identify the SVs related to the disease phenotype. DNA samples were sheared to 300 bp fragments using the Q800R sonicator (Covaris, United States), and a WGS library was constructed using the Illumina DNA PCR-Free Prep (Illumina). WGS was performed on the NovaSeq 6000 (Illumina) using 2 × 150 bp sequencing. Then, CNV-seq analysis was performed as previously described [19]. Whole-exome sequencing (WES) was performed to identify diseases causing single-nucleotide variants (SNVs) and short insertions and deletions (Indels) in the protein-coding regions of the genome. WES was performed using the xGen® Exome Research Panel v1.0 (IDT, USA) on the Illumina NovaSeq6000 (Illumina, San Diego, CA, United States). Trios-WES data were annotated according to our previously described bioinformatic pipelines [20].

This process may lead to the exclusion of recognized pathogenic mutations associated with cardiac problems, as all bioinformatic systems generate false negative results.The 2015 ACMG/AMP Standards and Guidelines were applied to assess the pathogenicity of the detected variants [21].

### Validation of a de novo variant

Sanger sequencing was performed on the family trio to validate the putative variants with pathogenicity. Supplementary Table S2 contains a list of primers used for Sanger sequencing and PCR amplification. The family trios were tested using droplet digital PCR (ddPCR) to exclude the potential parental somatic mosaicism of the putative variant with important implications for the recurrence risk. ddPCR experiments were performed using a Droplet Digital PCR XQ200 system (Bio-Rad, CA, USA) according to the manufacturer’s recommendations. Sequences of primers and locked nucleic acid probes are presented in Supplementary Table S2. Each 20 μL PCR reactions contained 10 μL of 2X ddPCR Supermix for probes (Bio-Rad), 1 μL of probes and primers mix, and 100 ng of DNA. Approximately 20 μL of PCR reactions were loaded in a droplet generator cartridge (Bio-Rad, CA, USA) and droplets were generated with 70 μl of droplet generation oil (Bio-Rad, CA, USA) using the Q×200Droplet Generator (Bio-Rad, CA, USA). Following droplet generation, ddPCR droplets were transferred to a 96-well PCR plate and amplified on a thermocycler using the following cycling conditions: one cycle at 95°C for 600 seconds, followed by 40 cycles at 94°C for 30 seconds and 60°C for 60 seconds, one cycle at 98°C for 600 seconds, and 4°C for an unlimited period. After thermal cycling, droplet counts were read using the Q×200Droplet Reader (Bio-Rad) and data were analyzed by Quantasoft version 1.7 Studio (Bio-Rad).

### Cloning and mutagenesis of HCN4

Expression vectors (pCDNA 3.1) containing either the wild type (WT) or C679Y mutant (mut) hHCN4 were constructed as follows. Using the human complementary DNA (cDNA) library as a template, PCR fragments containing WT hHCN4 combined with 3xflag-tag, and EcoRI, XhoI restriction cleavage sites were generated using the following primers: hHCN4 5’ AATTCATGGACAAGCTGCCGCCGTCC and hHCN4 3’ TCGAGTCATTTGTCGTCATCATCCTTATAGTCCTTATCATCGTCGTCTTTG

TAATCCTTGTCATCGTCATCCTTGTAGTCTAGATTGGATGGCAGTTTGG. Then, pCDNA 3.1 were digested with EcoRI and XhoI (Fermentas), and the digested products were ligated with PCR fragments using T4 ligase (Fermentas). The ligated product was introduced into *Escherichia coli* DH5α and the plasmid was extracted from ampicillin-resistant colonies. Point mut-plasmid pCDNA 3.1- G2036A hHCN4 was constructed using the PCR mut method. Briefly, using WT-plasmid pCDNA 3.1- hHCN4 extracted from overnight cultured *Escherichia coli* DH5α as a template, point mut plasmid was generated using the following primers: C679Y hHCN4 5’ ACACCTACTACCGCCTCTACTCGCTGAGCG and C679Y hHCN4A 3’ TAGAGGCGGTAGTAGGTGTCGGCCCTCACG. The PCR product was digested with DNase I (Fermentas), which only digested DNA from bacteria, leaving the newly synthesized point mut plasmids. All strains and plasmids were validated by DNA sequencing.

### Cell culture and transfection

Expression vectors (pCDNA 3.1- hHCN4 and pCDNA 3.1- G2036A hHCN4) and a vector containing a green fluorescent protein (pCDH-CMV-MCS-EF1-copGFP) were co-transfected in HEK293 cells using polyethylenimine transfection reagent.

### Voltage clamp recordings and data analysis

Whole-cell voltage clamping recordings were performed in HEK293 cells transiently expressing WT-HCN4 or mut-C679Y HCN4 channels together with GFP following a 24–48-hour transfection. The contents of the extracellular solution (in mM) included NaCl 110, MgCl_2_ 0.5, KCl 30, CaCl_2_ 1.8, and HEPES 5 (pH adjusted to 7.4 with NaOH). The contents of the pipette solution (in mM) were KCl 130, NaCl 10, MgCl_2_ 0.5, EGTA 1, HEPES 5, MgATP 2, NaGTP 0.1, and phosphocreatine 5 (pH adjusted to 7.4 with KOH). The cells used for patch clamp recordings were those exhibiting green fluorescence. The Axopatch 200B amplifier, the Digidata 1550B data acquisition device, and pCLAMP 10.6 software were used to capture data at room temperature. Various voltage protocols were implemented as previously mentioned [7,22] to assess the functional characteristics of HCN4 channels. In summary, voltage steps between −50 and −140 mV were used for activation characteristics, with the holding potential set at −40 mV, with an increment of 10 mV. The length of the negative voltage steps decreased from 24 seconds (−50 mV) to 3 seconds (−140 mV) with an increase in hyperpolarization. Deactivation properties were evaluated using a paired-pulse technique from a holding potential of −40 mV. Two pulses were applied: one at −120 mV and the other at increasing intervals of 1–7 seconds.

The relationship between current and voltage (I-V), as well as the reversal potential (Erev), were established using hyperpolarizing pulses (−120 mV; 3 seconds), followed by depolarizing voltage steps ranging from −120 mV to 20 mV. The half-maximal activation voltages (V_1/2_) and slope factors (κ) of the activation curves were determined by fitting normalized data to a Boltzmann equation (y=A2+(A1-A2)/(1+exp((x-x0)/dx))) [23]. The reversal potential was computed utilizing a third-order polynomial function [22,24].

### Statistical analysis

All data were expressed as the means ±SEM. Comparisons between groups for continuous variables were conducted using either the student’s t-test. GraphPad Prism5.0 was used for statistical analyses. Statistical significance was defined as *p* < 0.05.

## Supplementary Material

Supplementary material .doc

## Data Availability

The raw sequence data reported in this paper have been deposited in the Genome Sequence Archive in National Genomics Data Center, China National Center for Bioinformation/Beijing Institute of Genomics, Chinese Academy of Sciences (https://ngdc.cncb.ac.cn/gsub/, reference number [subHRA012410].). The supplemental vide is available at https://doi.org/10.57760/sciencedb.21622. After the publication of the study findings, the data will be available for others to request. The corresponding author (feng zhu) will provide the accession number of the database once the data are approved to be shared with others. The clinical data cannot be shared for privacy concerns.
