# Supplementary material for: A rare HCN4 variant combined with sick sinus syndrome, left ventricular noncompaction, and complex congenital heart disease
Source: Channels (Austin). 2025 Jul 4;19(1):2517851. doi: 10.1080/19336950.2025.2517851 (PMC12233691; doi:10.1080/19336950.2025.2517851)
Supplement: Supplementary material .doc [file KCHL_A_2517851_SM1722.doc]

**Supplemental video:** **The transthoracic ultrasound of the heart showed** **malformation of** **quadricuspid aortic valve (QAV)（https://www.bilibili.com/video/BV1LDPYeXED4/）.**

**Supplemental Table 1 The number of variants after each filtering step**

|  | **Variants numbers** |
| --- | --- |
| **Filtering Step** | **Fetus** |
| Initial | 89,021 |
| 1. exclude the variants outside exonic and splicing regions | 26,707 |
| 2. exclude the variants with MAF* > 0.01 | 1,426 |
| 3. exclude the synonymous variants in exome | 959 |
| 1. Include the variants inherited in *de novo*, AR and XR patterns# | 215 variants in 75 genes |

*MAF:Minor Allele Frequency in gnomAD, 1000 genome and Exome Aggregation Consortium database

# AR, autosomal recessive;XR, X-linked recessive.

**Supplementary table 2 Primer and probe sequences**

| Primer information | Primer and probe sequences |
| --- | --- |
| ddPCR primers amplfying a fragment containing a HCN4 variant | 5′-CACCTCATTGAAGTTGTCCACG−3′(forward)  5′-TCCTGATGCTCTGGCAGAGA−3′(reverse) |
| mutant specific (FAM-labelled) and wildtype specific (VIC-labelled) locked nucleic acid probes for ddPCR | 5′-(FAM)CGAGTAGAGGCGGCAGTA−3′(mutant)  5′-(VIC)CGAGTAGAGGCGGTAGTA−3′(wildtype) |
| Primers for Sanger sequencing | 5′-CACCTCATTGAAGTTGTCCACG−3′(forward)  5′-TCCTGATGCTCTGGCAGAGA−3′(reverse) |

FAM, fluorescent dye 5-Carboxyfluorescein;

VIC, fluorescent dye 2’-chloro-7’-phenyl-1,4-dichloro-6-carboxyfluorescein.
